# Ecological Impacts of Mining in the Amazon: Thematic Trends and Research Gaps

**DOI:** 10.1007/s00267-026-02403-6

**Published:** 2026-02-20

**Authors:** Jonison Vieira Pinheiro, Walmer Bruno Rocha Martins, Juliana Siqueira-Gay, Sara Villén-Pérez, Vinicius José Giglio

**Affiliations:** 1 Universidade Federal do Oeste do Pará, Campus Oriximiná, Oriximiná, PA Brazil; 2 Universidade Federal Rural da Amazônia, Campus Capitão Poço, Rua Profa Antônia Cunha de Oliveira, Capitão Poço, PA Brazil; 3 Universidade de São Paulo, Escola Politécnica, São Paulo, SP Brazil; 4 Universidad de Alcalá, GloCEE – Global Change Ecology and Evolution Research Group, Department of Life Sciences, Alcalá de Henares, Comunidad de Madrid Spain

## Abstract

Mining activities have expanded rapidly in the Amazon, generating ecological, social, and health concerns. Although the number of publications addressing mining impacts has grown, studies often lack integrative assessments. Here, we provide a systematic synthesis of how the ecological impacts of mining in the Amazon have been framed in the scientific literature over the last 30 years (1995–2025). We analysed 462 peer-reviewed articles and applied Latent Dirichlet Allocation (LDA) to identify the main themes discussed in the literature and the research gaps that structure this field. Twelve topics emerged and were grouped into five categories: Pollution and Toxicology, Land Use, Social–Ecological Systems, Biodiversity and Ecosystem Health, and Monitoring and Assessment. The most prevalent topics were mercury bioaccumulation, human mercury exposure, and post-mining restoration, highlighting persistent concerns with toxicological pathways and ecosystem recovery. In contrast, environmental monitoring & impact indicators, disease hotspots, and landscape changes due to resource extraction were the least represented, revealing limited attention to broader-scale ecological processes and early-warning indicators. Semantic similarity analysis showed close relationships among topics linked to contaminant pathways and human health, as well as between landscape alteration and stream biodiversity. The co-occurrence–based dissimilarity analysis revealed weak connections among several topic pairs and highlighted substantial gaps, particularly the infrequent association between monitoring indicators and either toxicological or biodiversity-focused studies. Together, these findings reveal structural imbalances in the scientific agenda. Bridging ecological, toxicological, and socio-political perspectives is essential to support evidence-based responses and safeguard both biodiversity and human well-being in the Amazon.

## Introduction

The Amazon basin, the largest tropical forest and most biodiverse biome on Earth (Guayasamin et al. 2024), has experienced a rapid expansion of mining activities over the past few decades (Lobo et al. 2016; Kalamandeen et al. 2020; Siqueira-Gay and Sánchez 2021; MapBiomas 2024; Cortinhas Ferreira Neto et al. 2024). Both large-scale industrial operations and artisanal and small-scale gold mining have increased land-use pressures, often occurring outside regulatory frameworks (Machado and Figueirôa 2001; Kalamandeen et al. 2020; Bruno et al. 2020). Mining in this region is linked to many ecological impacts, including deforestation and biodiversity loss (Sonter et al. 2017), habitat degradation (Santana et al. 2020; Meißner 2021), soil erosion (Angon et al. 2024), metal contamination and bioaccumulation in food webs (Souza et al. 2025). These impacts are further compounded by the socio-economic context, as mining activities frequently overlap with Indigenous territories, local communities, and areas of high ecological sensitivity, raising concerns about both ecosystem integrity and human well-being (Silva et al. 2023).

Despite the growing number of studies addressing mining in the Amazon, research on its ecological impacts remains fragmented. Most studies focus on specific dimensions such as heavy metal contamination (e.g., de Vasconcellos et al. 2022; Martoredjo et al. 2024; Angon et al. 2024), forest loss (e.g., Sonter et al. 2017; Caballero Espejo et al. 2018; Siqueira-Gay and Sánchez 2021), or post-mining restoration (e.g., Martins et al. 2022; Barbosa et al. 2022), and rarely integrate multiple scales, temporal dynamics, or socio-ecological interactions. This fragmentation limits our understanding of the cumulative and interconnected consequences of mining and hinders the development of holistic strategies for environmental management and policy-making. A systematic synthesis of this literature is therefore essential to clarify research trends, identify knowledge gaps, and provide a foundation for evidence-based decision-making.

Recent advances in computational approaches offer powerful tools for synthesizing large and complex scientific literatures. Topic modeling, and in particular Latent Dirichlet Allocation (LDA), identifies co-occurring themes within texts, revealing patterns, structures, and trends that may not be apparent through traditional narrative reviews (Blei et al. 2003; Blei 2012). By capturing thematic distributions across decades, disciplines, and methodological approaches, LDA allows researchers to detect dominant topics, underexplored areas, and relationships between research themes, providing a more integrative understanding of how ecological impacts are studied.

Here, we applied topic modeling to a corpus of 462 peer-reviewed articles published between 1995 and 2025 to investigate how the ecological impacts of mining in the Amazon have been discussed in the scientific literature. Although artisanal and small-scale mining activities intensified in the Amazon in the 1980s, particularly in Brazil, our preliminary survey indicated that articles addressing their ecological impacts only gained broader visibility and consistency from the mid-1990s onward. Consequently, we began our review in 1995, when the literature had become more consistent in capturing mining-related ecological concerns. Specifically, our study addresses four questions: (i) What are the main research themes concerning mining-related ecological impacts discussed in the Amazonian context? (ii) How are these themes distributed across broader categories? (iii) Which topics are most prevalent? (iv) Which themes frequently co-occur? By addressing these questions, this study provides a structured overview of the field, identifies persistent knowledge gaps, and offers a roadmap to guide future research and conservation strategies in one of the world’s most critical and vulnerable biomes.

## Materials and Methods

### Data Collection

We assessed articles from the Web of Science, SciElo, and Scopus databases to ensure comprehensive coverage of the peer-reviewed literature on the topic. Although searches were not restricted by language, all retrieved documents contained English abstracts, even when the full texts were written in Portuguese or Spanish. Thus, all titles and abstracts were processed in English for subsequent text-mining and topic modeling. To compile these scientific articles, we applied database-specific search strings to titles, abstracts, and author keywords, combining terms related to mining activities and ecological impacts in the Amazon. The complete search operators and query structures used in each database are provided in the Supplementary Material (SM1 Table S1). No filter was applied during the search process. The derivation of the term mining in Portuguese and Spanish was included because some studies employ these words even in English texts. The use of truncation (e.g., the asterisk symbol *) ensured the retrieval of word variations, including those with diacritics. We included original research articles published between January 1, 1995, and May 13, 2025, which corresponds to the date of the last search.

**Fig. 1:**
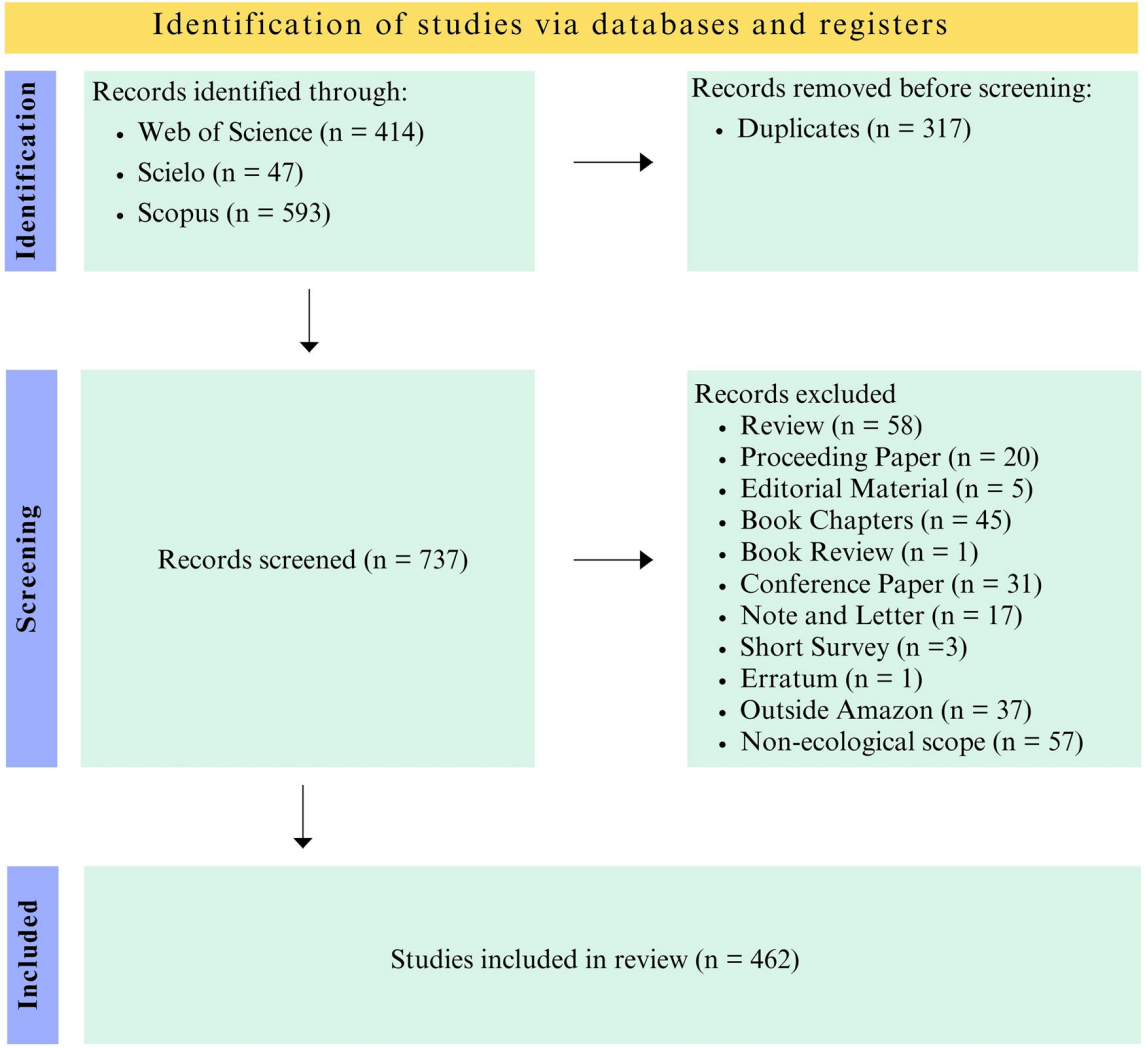
Flow diagram illustrating the identification, screening, and selection of studies on the ecological impacts of mining in the Amazon. The diagram details the number of records identified, excluded, and retained at each stage, from initial database searching to the final set of studies included in the analysis

The initial search yielded a total of 1,054 records (414 from Web of Science, 593 from Scopus, and 47 from SciELO). After excluding non-article types (such as book chapters, conference papers, remaining reviews, notes, and letters), 873 articles remained, of which we excluded 57 studies that did not address ecological impacts, 37 conducted outside the Amazon region, and 317 duplicates. A total of 462 articles were included in the final analysis, all of which discussed the ecological impacts of mining activities in the Amazon region (Fig. 1). The complete list of articles included in the analysis is available as Supplementary Material (SM1 Table S2). All documents included in the analysis were imported into R software v. 4.5.2 (R Core Team 2025) for topic modeling.

### Data Preprocessing

The research content was created by combining texts extracted from article titles and abstracts. Due to the large amount of irrelevant information in the unstructured data, it was necessary to preprocess (i.e., clean) the data prior to model training and subsequent analyses. Using the R package *tm* (Feinerer and Hornik 2023), raw text was tokenized, that is, the sentences were transformed into individual words, also known as unigrams. Subsequently, the text was transformed to lowercase, and all whitespace, irrelevant characters, digits, and stopwords (i.e., ‘the’, ‘that’, ‘this’) were removed using the built-in stopword lists. In addition, a customized list of stopwords specifically adapted for this study was applied (see SM1 Table S3). To standardize inflected words, the remaining text was stemmed using the *stemDocument()* function from the *tm* package, which applies the Snowball stemming algorithm (e.g., “contaminated” → “contaminate”). However, to preserve contextual meaning, bigrams and trigrams (e.g., “ecological_impact”, “indigenous_land_rights”) were previously identified and preserved using the *ngram* package (Schmidt and Heckendorf 2023).

Tokens were counted at each step of preprocessing: 113,727 tokens in the raw text, 62,133 tokens after cleaning (lowercasing, removal of stopwords, digits, whitespace, and irrelevant characters), and 45,617 tokens in the Document-Term Matrix (after removing very rare terms with a frequency < 10 and very common terms with a frequency > 617). These thresholds were selected after inspecting the empirical term-frequency distribution and validated through a sensitivity analysis in which we systematically varied both lower-frequency and upper-frequency limits (rare-term thresholds from 1 to 17; common-term thresholds of 387, 403, 428, 581, and 617). Across all combinations, the number of retained terms and total tokens remained highly stable (SM1 Table S4), indicating that the chosen cutoffs (< 10 for rare terms and > 617 for very common terms) effectively reduce noise while preserving the informative structure of the vocabulary. This refinement is important because rare terms tend to introduce noise due to their low representativeness, whereas very common terms often lack semantic specificity and contribute little to topic differentiation (Silva and Ribeiro 2003; Westgate et al. 2015).

### Topic Modelling

We used the Latent Dirichlet Allocation (LDA) model (Blei et al. 2003) to analyze the content of the retrieved articles. LDA treats documents as mixtures of topics in different proportions. For example, an article can be 50% about “water pollution”, 30% about “soil pollution”, and 20% about “biodiversity loss”. A topic is described as a set of words that frequently appear together, providing an understanding of the underlying themes within a corpus (Luiz et al. 2019).

The optimal number of topics (*k*) was determined using the coherence scores calculated using the Deveaud2014 metric (Deveaud et al. 2014). Because coherence alone does not guarantee that a topic solution is robust across different initializations, we additionally evaluated topic stability. For this purpose, we re-estimated the LDA model with 20 different random seeds, each producing an independent Gibbs-sampled solution with the same *k* value. We then quantified the similarity between the resulting topic-term distributions using the Jaccard coefficient computed on the top terms of each topic. Together, the coherence maximization and the stability assessment provided complementary evidence supporting the choice of *k* as both statistically sound and substantively meaningful for this corpus. Each topic was initially labeled based on its 30 most probable words. The proposed labels were then validated by reviewing the abstracts of the articles in which each topic was most prevalent. Topic names were independently reviewed by authors, four experts in the field of ecology, to ensure accuracy and consistency. Any discrepancies were resolved through discussion until consensus was reached.

### Prevalence of Topics in the Corpus

To evaluate how topics are distributed across the corpus, we identified the dominant topic in each document based on the highest posterior topic probability from the LDA model. Using these dominant assignments, we calculated both the number of documents (n) and the percentage (%) of the corpus associated with each topic.

To quantify uncertainty around these estimates, we generated 95% confidence intervals via bootstrapping, resampling documents with replacement 1,000 times and recalculating topic prevalence in each iteration. This approach provided a robust measure of topic prominence in the dataset, indicating which themes appear most frequently as the primary focus of individual studies. The results were visualized as a horizontal bar chart.

### Topic Similarity

To assess semantic similarity among topics, we used the word–topic probability matrix (β matrix), which represents each topic as a probability distribution across the vocabulary. The matrix was subjected to a Hellinger transformation to account for the compositional nature of the data and to reduce the influence of highly frequent terms. Pairwise Euclidean distances were then computed between topics using the transformed matrix, providing a measure of how similar their word distributions are. The resulting distance matrix was visualized through non-metric multidimensional scaling (NMDS) using the *metaMDS* function from the *vegan* package (Oksanen et al. 2025), which allowed us to represent the multivariate structure in a reduced dimensional space.

### Research Gap Analysis

To identify research gaps, we quantified the dissimilarity between topics using the document–topic probability matrix (W-matrix) (SM1 Table S5), where rows represent documents and columns represent topic probabilities. The matrix was transposed so that each row corresponded to a topic, allowing pairwise dissimilarities to be computed. Euclidean distances were then calculated between topics, providing a measure of how differently they are distributed across the document corpus. The resulting distance matrix was rescaled to a 0–1 interval to facilitate visualization. A heatmap was generated to display these dissimilarities, with higher values indicating topic pairs that co-occur infrequently across documents, thus highlighting potential research gaps.

### Temporal Analysis of Topic Prevalence

To evaluate temporal changes in topic prevalence, we used the W-matrix. For each document, topic proportions (representing the probability assigned to each topic) were linked to their corresponding publication year. The dataset was first transformed from wide to long format, resulting in a structure where each row represented a single topic proportion associated with a given document and year. We then aggregated these values to obtain the mean prevalence of each topic per year. This was accomplished by grouping the long-format data by publication year and topic and calculating the mean topic proportion for each combination. The resulting summary dataset contained yearly averages for all topics across the entire study period.

## Results

The coherence analysis indicated that *k* = 12 was the optimal number of topics, as it exhibited the highest Deveaud2014 coherence score among all tested values (see SM1 Table S6, and SM2 Fig. S1). Pairwise comparisons of the resulting topic-term structures using the Jaccard similarity index revealed a mean similarity of approximately 0.4305, indicating a moderate but systematic convergence of topic composition across replications (SM1 Table S7).

**Fig. 2:**
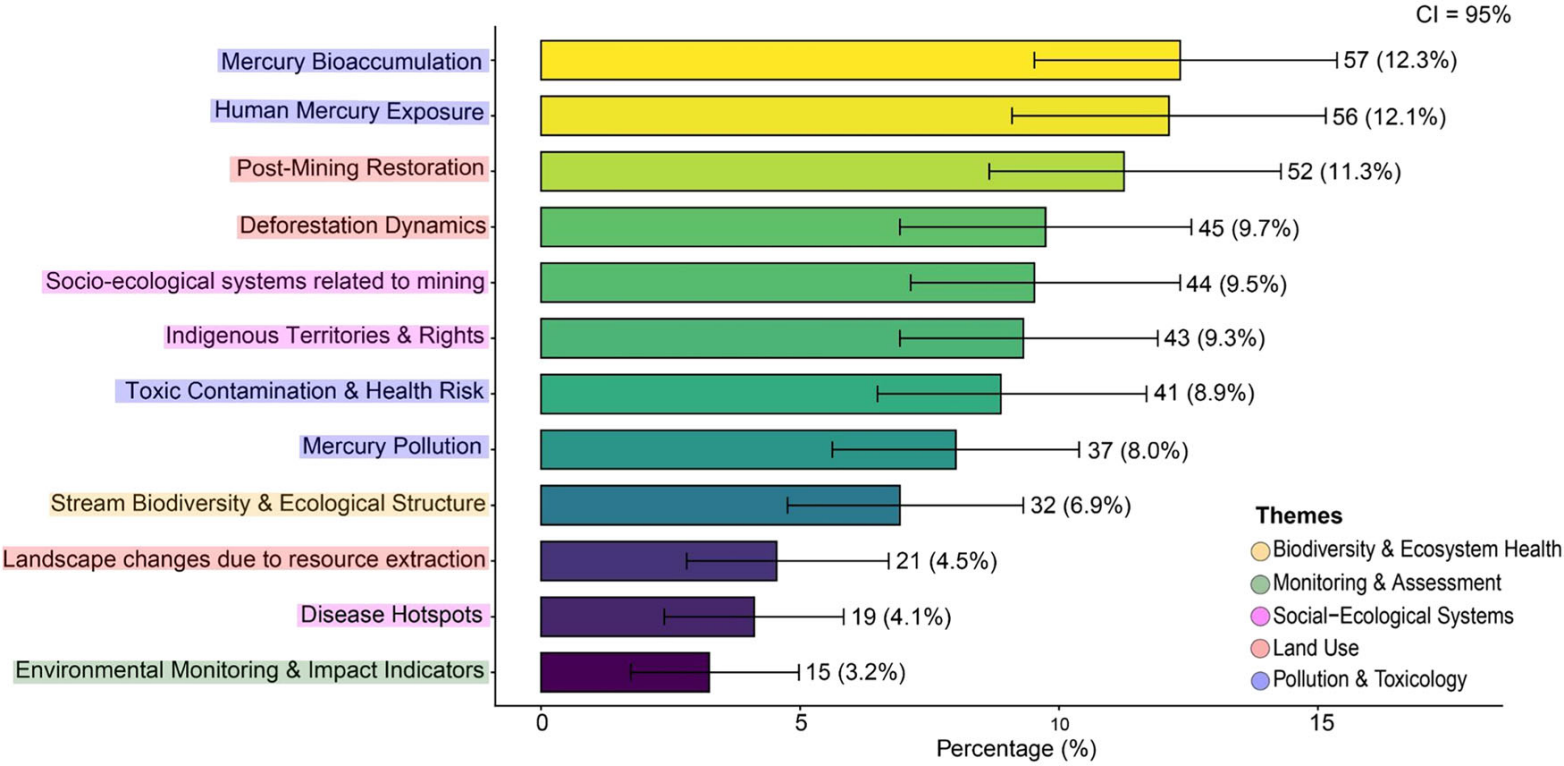
Distribution of dominant topics across the corpus of 462 articles discussing the ecological impacts of mining in the Amazon. Each bar represents a topic, with the label indicating both the number of documents (*n*) and the corresponding percentage (%) in which the topic is dominant according to the LDA model. The color gradient encodes topic prevalence, with lighter (yellowish) tones representing higher prevalence and darker tones lower prevalence. Error bars show the 95% confidence intervals (CI = 95%) estimated via bootstrapping

The topics were grouped into five thematic categories based on content: (1) Pollution & Toxicology (Mercury bioaccumulation, Toxic contamination & health risk, Mercury pollution, Human mercury exposure); (2) Land Use (Post-mining restoration, Deforestation dynamics, Landscape changes due to resource extraction); (3) Social–Ecological Systems (Indigenous territories & rights, Socio-ecological systems related to mining, Disease hotspots); (4) Biodiversity & Ecosystem Health (Stream biodiversity & ecological structure); and (5) Monitoring & Assessment (Environmental monitoring & impact indicators). Most of the topics were related to the theme Pollution & Toxicology (*n* = 4), followed by Social–Ecological Systems (*n* = 3) and Land Use (*n* = 3) (Table 1, but see Fig. S2 and Table S8 for a detailed visualization of probability distributions of the top ten words per topic and the most representative articles for each topic). The most prevalent topics were *Mercury bioaccumulation* (12.3%), *Human mercury exposure* (12.1%), and *Post-mining restoration* (11.3%). The least representative topics were *Environmental monitoring & impact indicators* (3.2%), *Disease* *hotspots (*4.1%), and *Landscape changes due to resource extraction* (4.5%) (Fig. 2).

**Table 1:** Uncovered topics from 462 research articles about mining in the Amazon published between 1995 and 2025 identified using Latent Dirichlet Allocation Modelling (LDA)

Topic	Label	Top topic words	Theme	Description
1	Mercury bioaccumulation	river, water, fish, sediment, concentr, site, hg_concentr, lake, season, show, sourc, basin, increas, river_basin, flood, rang, contamin, freshwat, amazon_basin, mercuri, distribut, affect, anthropogen, system, part, period, upper, madeira_riv, mug_g, tapajo	Pollution & Toxicology	Addresses the environmental and ecological impacts of mercury contamination associated with gold mining activities. It examines mercury bioaccumulation across various aquatic compartments, including water, sediments, plankton, and fish.
2	Toxic contamination & health risk	contamin, risk, concentr, valu, potenti, sampl, pollut, higher, collect, area, content, metal, level, tail, human_health, wast, environment, element, evalu, heavy_met, organ, indic, deposit, analyz, compar, amazon_brazil, lead, tissu, muscl, differ	Pollution & Toxicology	Assesses contamination by toxic substances and the resulting risks to human and environmental health.
3	Stream biodiversity & ecological structure	speci, communiti, differ, environment, stream, influenc, indic, import, function, divers, ecolog, habitat, structur, aquat, site, composit, pattern, alter, disturb, assemblag, eastern, higher, record, variabl, sampl, chang, exhibit, determin, tempor, local	Biodiversity & Ecosystem Health	Analyzes ecological community structure and biodiversity patterns in aquatic habitats under mining stress.
4	Post-mining restoration	soil, forest, plant, restor, speci, recoveri, growth, degrad, rehabilit, chemic, ecosystem, year, differ, fertil, site, nativ, densiti, process, tree, biomass, treatment, evalu, effici, organic_matt, pit, veget, initi, nutrient, stage, abandon	Land Use	Focuses on the recovery of soil properties in post-mining landscapes. It includes the use of organic and chemical amendments to improve soil quality and support the reestablishment of vegetation after mining extraction.
5	Landscape changes dueto resource extraction	area, studi, caus, time, analysi, environ, veget, mining_act, type, mining_area, index, locat, detect, imag, provid, process, data, known, similar, sand, year, map, cover, tropic, canga, conduct, surfac, open, monitor, reveal	Land Use	Discusses changes in land cover and land use over time, including monitoring environmental alterations caused by human activities such as mining, agriculture, and infrastructure development.
6	Mercury pollution	gold_min, mercuri, gold, asgm, small_scal, artisan, activ, peru, peruvian_amazon, impact, emiss, estim, follow, amalgam, atmospher, madre_dio, factor, find, mercury_pollut, surinam, elev, previous, region, histori, inform, per, releas, global, increas, produc	Pollution & Toxicology	Focuses on mercury pollution from artisanal and small-scale gold mining, linking historical legacies with current environmental and health risks.
7	Indigenous territories & rights	state, extract, brazilian, land, govern, indigen, illeg, polici, peopl, nation, protect, research, indigenous_peopl, resourc, unit, action, new, current, zone, intern, communiti, right, case, territori, threat, world, exploit, legal, vulner, exist	Social-Ecological Systems	Discusses land conflicts and governance issues concerning Indigenous communities affected by mining.
8	Environmental monitoring & impact indicators	impact, assess, activ, relat, human, respons, contribut, identifi, effect, consid, system, studi, includ, environmental_impact, evalu, associ, indic, affect, analyz, approach, appli, ecuador, integr, locat, greater, monitor, research, carri, ecosystem, essenti	Monitoring & Assessment	Addresses how environmental monitoring and impact indicators are used to assess both the human health consequences of environmental degradation and the effectiveness of ecological restoration techniques in mining areas.
9	Human mercury exposure	level, fish, exposur, popul, mercuri, health, mean, mu_g, hair, children, associ, methylmercuri, signific, mehg, communiti, studi, fish_consumpt, mercury_exposur, age, year, expos, rang, sampl, effect, live, test, food, limit, non, total_mercuri	Pollution & Toxicology	Focuses on studies assessing human exposure to mercury through fish consumption, with a focus on health effects in vulnerable populations such as children and riverine communities.
10	Socio-ecological systems related to mining	develop, environment, econom, social, miner, local, urban, sustain, industri, manag, natur, product, project, process, brazil, model, oper, understand, articl, propos, sector, global, plan, generat, communiti, growth, practic, address, cost, rural	Social-Ecological Systems	Examines the social perception, acceptance, and impacts of mining projects, particularly regarding how local communities interact with, depend on, and respond to ecological changes driven by mining activities within social–ecological systems.
11	Deforestation dynamics	deforest, forest, chang, increas, region, conserv, biodivers, land_us, agricultur, loss, expans, reduc, spatial, futur, rate, impact, protected_area, log, decad, effect, carbon, model, dynam, annual, develop, scenario, signific, occur, trend, climat	Land Use	Covers deforestation trends, focusing on land use change, climate impacts, and associated health and carbon cycle consequences using spatial modeling.
12	Disease hotspots	brazil, amazon, present, region, studi, brazilian_amazon, low, popul, analysi, control, work, rate, possibl, import, problem, addit, measur, point, number, diseas, malaria, repres, condit, municip, intens, biolog, report, world, dam, select	Social-Ecological Systems	This topic covers studies identifying spatial patterns, high-risk areas, and environmental and socio-economic factors driving disease occurrence, particularly malaria.

The topics present the thirty most probable words, the assigned label, the thematic classification and a brief description of each topic.

**Fig. 3:**
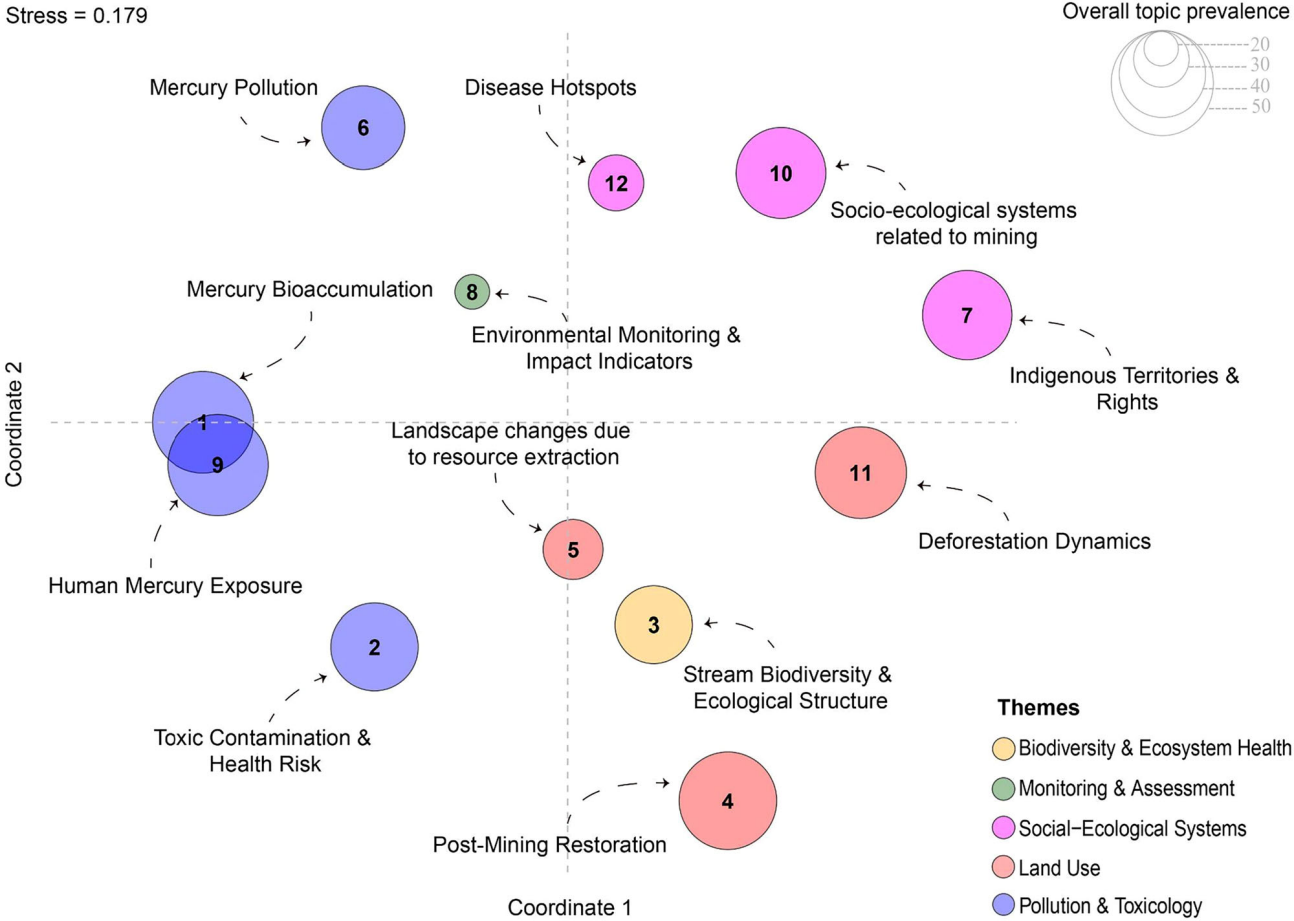
Intertopic distance map based on non-metric multidimensional scaling (NMDS) of topic-word distributions. Each bubble represents a topic, with distances reflecting word similarity between topics. The bubble size indicates overall topic prevalence, and colors denote thematic categories

The semantic similarity analysis revealed proximity between the topics *Mercury bioaccumulation* and *Human mercury exposure*, *Landscape changes due to resource extraction* and *Stream biodiversity & ecological structure*, and finally between *Disease hotspots* and *Socio-ecological systems related to mining* (Fig. 3).

**Fig. 4:**
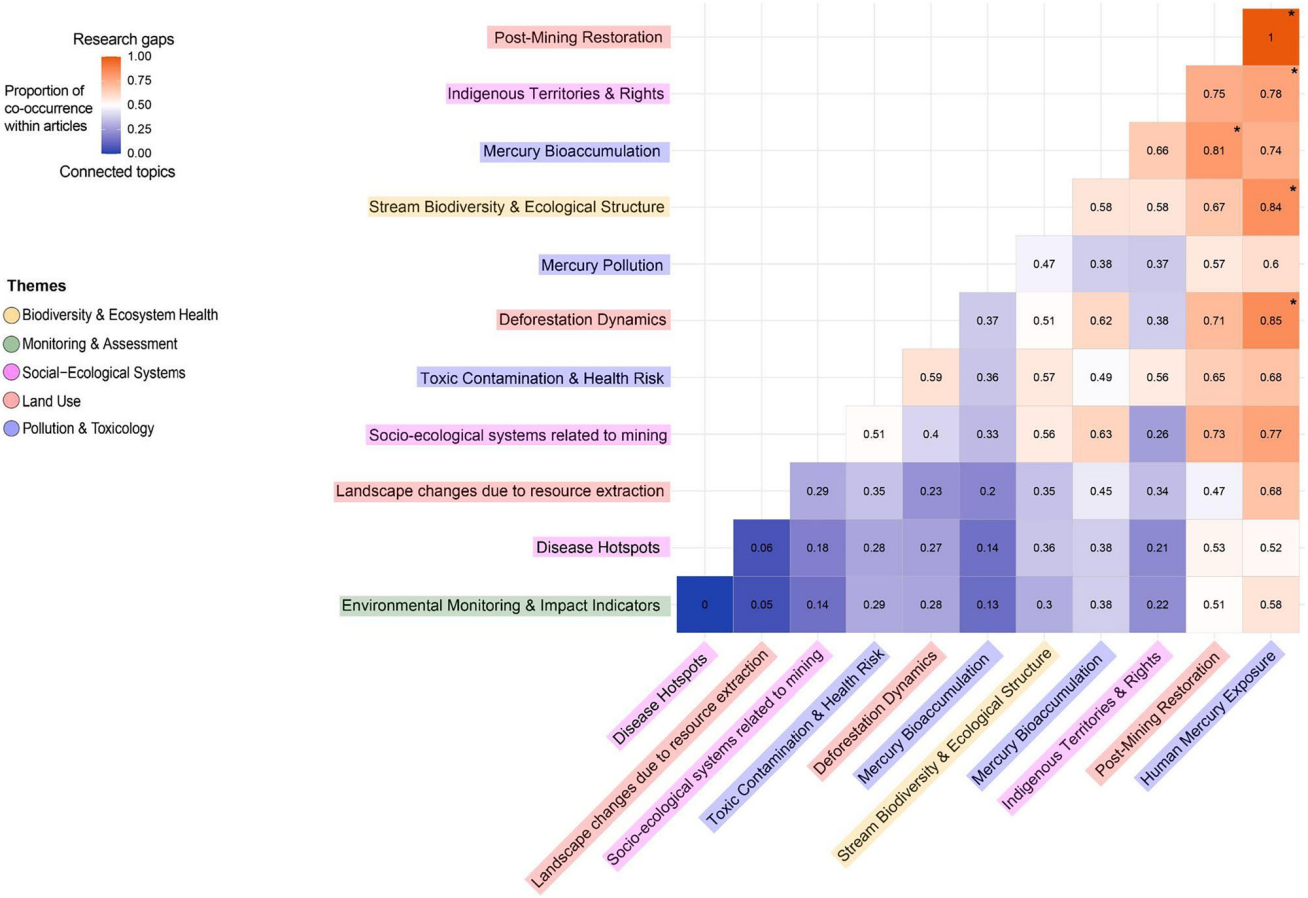
Research gaps in the literature on the ecological impacts of mining in the Amazon. The heatmap displays the dissimilarity values derived from the topic–per–article proportion distance matrix, with each square indicating how rarely pairs of topics are studied together. Higher values represent larger research gaps. The color gradient ranges from red (high dissimilarity; topics rarely examined together) to blue (low dissimilarity; more frequently connected topics). Squares marked with an asterisk identify the five highest dissimilarity values, highlighting the most pronounced gaps. The colored labels indicate the thematic category assigned to each topic

A high dissimilarity was observed between topics, such as *Post-mining restoration* and *Human mercury exposure* (dissimilarity = 1), *Human mercury exposure* and *Deforestation dynamics* (dissimilarity = 0.85), *Human mercury exposure* and *Stream biodiversity & Ecological structure* (dissimilarity = 0.84), *Mercury bioaccumulation* and *Post-mining restoration* (dissimilarity = 0.81), *Indigenous territories & rights* and *Human mercury exposure* (dissimilarity = 0.78), *Socio-ecological systems related to mining* and *Human mercury exposure* (dissimilarity = 0.77) (Fig. 4).

**Fig. 5:**
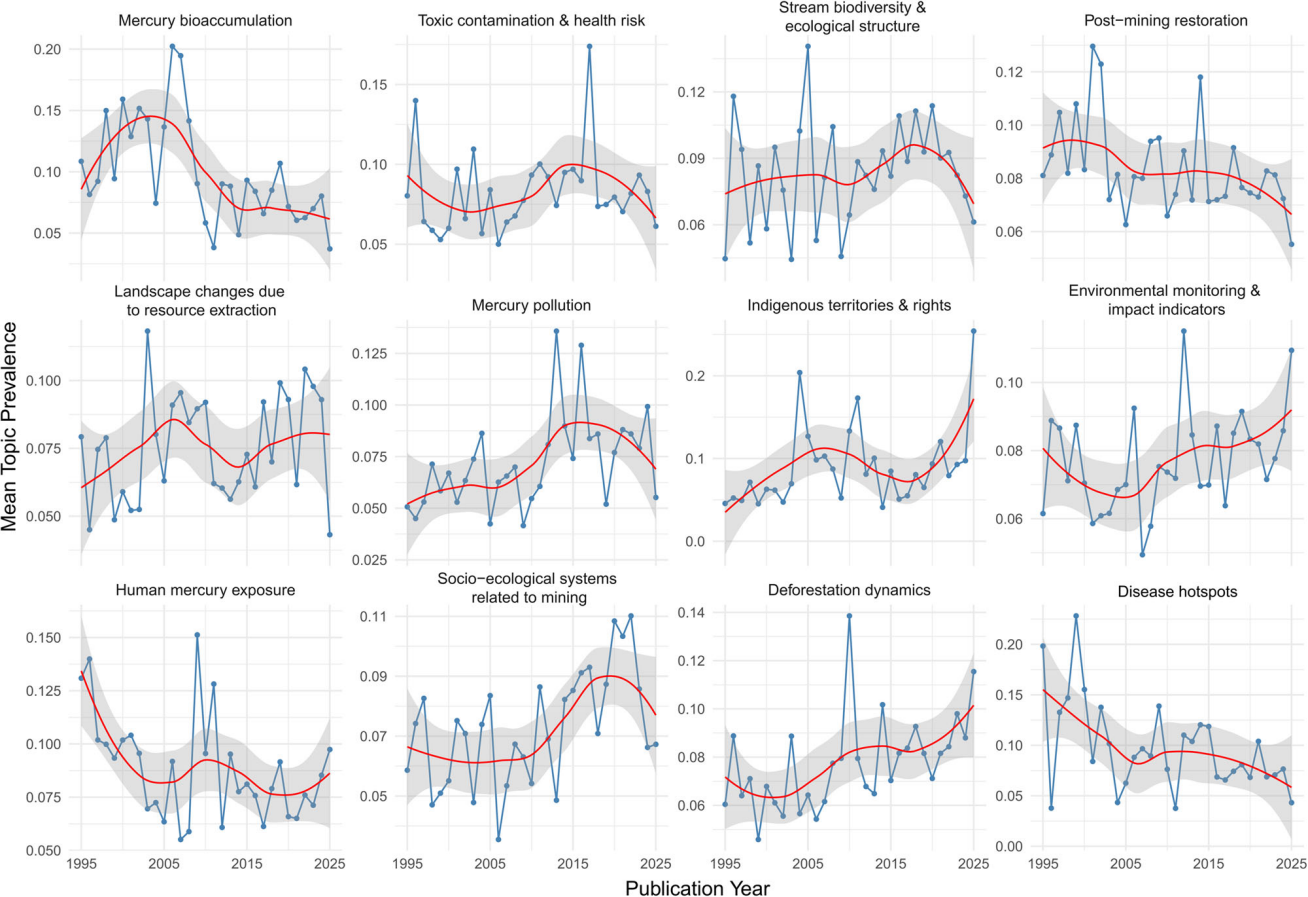
Temporal dynamics of mean topic prevalence from 1995 to 2025. Each panel shows the annual mean prevalence of a specific topic, based on document-level proportions. Points represent yearly averages, the blue line shows the observed trend, and the red LOESS curve illustrates the smoothed temporal trajectory. The grey band represents the 95% confidence interval around the LOESS estimate

We observed distinct temporal patterns across topics, with some showing steady increases while others remained relatively stable or displayed more irregular fluctuations (Fig. 5). Among these patterns, several topics exhibited particularly pronounced temporal shifts. *Mercury bioaccumulation* showed a consistent decline, with mean prevalence decreasing from approximately 0.10 in 1995 to about 0.05 in 2025. *Human mercury exposure* peaked around 1995 ( ≈ 0.14) and declined gradually thereafter, reaching roughly 0.08 by 2025. *Post-mining restoration* also displayed a downward trend, decreasing from around 0.10 in the mid-1990s to approximately 0.06 in 2025.

*Toxic contamination & health risk* exhibited a more irregular temporal profile: mean prevalence started near 0.10, declined around the mid-2000s, increased again in subsequent years, and ultimately decreased toward 0.07 by 2025. In contrast, *Indigenous territories & rights* showed a substantial increase over the study period, rising from about 0.05 in 1995 to nearly 0.17 in 2025. *Disease Hotspots* demonstrated a declining trend, with mean prevalence falling from roughly 0.15 in 1995 to approximately 0.06 in 2025. The topic *Socio-ecological systems related to mining* increased from around 0.06 in the mid-2000s and reached its highest values between 2013 and 2015 ( ≈ 0.09). Finally, *Deforestation dynamics* increased gradually, rising from about 0.07 in 1995 to approximately 0.10 in 2025.

## Discussion

Our analysis provides an overview of the main themes and research gaps surrounding the ecological impacts of mining in the Amazon, revealing a structured yet uneven scientific landscape. Research in this field remains strongly oriented toward mercury contamination and ecosystem recovery, reflecting long-standing concerns over toxicological pathways and their consequences for food webs and local communities, i.e., mercury mobilization, bioaccumulation, and their cascading effects on food webs and local communities (Mestanza-Ramón et al. 2023; Martoredjo et al. 2024; Domingues et al. 2024; Paiva et al. 2025; Wasserman et al. 2025). In contrast, socio-ecological dimensions, such as disease processes, landscape changes, and environmental monitoring, receive comparatively limited attention, indicating persistent gaps in system-level understanding and highlighting the need for more integrative monitoring frameworks that move beyond contaminant-centric approaches.

### Integrating Monitoring Frameworks and System-Level Indicators

Advancing this area will require coordinated schemes that combine sentinel taxa, multi-metric bioindicator panels, and remote-sensing–based assessments of land transformation, enabling early detection of cumulative impacts and tighter integration between ecological, social, and health-related dynamics in mining-affected regions. Importantly, the establishment of long-term monitoring programs is essential to capture temporal trends and delayed ecological responses that are often missed by short-duration studies, thereby providing a more complete basis for assessing progressive mining-related disturbances. The scarcity of monitoring-focused studies is particularly critical, given that mining-induced disturbances often manifest gradually and across multiple ecological scales (i.e., environmental degradation, including soil erosion, water pollution, regional deforestation, and biodiversity loss) (Sonter et al. 2017; Lee et al. 2025).

The prominence of mercury-related topics is not surprising, as artisanal and small-scale gold mining has been historically identified as a major source of mercury pollution in tropical rivers, with well-documented consequences for both aquatic ecosystems and local populations (Bruno et al. 2020; Montaña et al. 2021; Aldous et al. 2024). In the Amazon, mercury concentrations have been found to exceed safety thresholds at various levels of the trophic cascade (Bastos et al. 2015; Martoredjo et al. 2024), with severe consequences for children’s development (Mendes et al. 2020). Such focus reflects a convergence between ecological and social priorities, as mercury contamination transcends disciplinary boundaries by simultaneously threatening biodiversity (Kumar et al. 2023), food security (Hacon et al. 2020), and human health (Charkiewicz et al. 2025).

The semantic similarity analysis indicates that the topics *Mercury bioaccumulation* and *Human mercury exposure* cluster closely, reflecting the strong conceptual coupling between ecotoxicological pathways and human health risks documented in Amazonian mining regions. Likewise, the proximity between *Landscape changes due to resource extraction* and *Stream biodiversity & Ecological structure* suggests that studies frequently link mining-driven habitat alteration, such as changes in water chemistry, sediment load, and channel morphology, to shifts in community composition and ecosystem structure in Amazonian streams (e.g., Ferreira et al. 2024). Finally, the similarity between *Disease hotspots* and *Socio-ecological systems related to mining* highlights research intersections where environmental degradation, contamination risks, and social vulnerability converge within mining territories. Taken together, these semantic linkages show that, although the topics were modeled as distinct, they gravitate around two major integrative axes in the literature, the contaminant exposure and human health, and the landscape transformation and ecological responses.

While semantic similarity does not imply causal linkage between the phenomena themselves, the clustering of these topics reveals how the scientific community tends to frame interconnected problems. In essence, the way words are used mirrors the way knowledge is organized: where terminologies converge, research fields often overlap, reinforcing certain narratives and, at times, leaving others unexplored. Recognizing these semantic proximities is therefore not merely descriptive; it identifies potential bridges for interdisciplinary collaboration and reveals where thematic silos might be broken to foster a more integrated understanding of mining’s ecological and social consequences in the Amazon.

### Thematic Linkages and Conceptual Divergence Among Topics

The research gap analysis revealed a markedly fragmented structure in the literature, but the meaning of this fragmentation is not uniform across topics. Some distances likely reflect expected conceptual boundaries, for example, *Post-mining restoration* and *Human mercury exposure* operate on distinct temporal and disciplinary axes, and their separation is consistent with the way these fields traditionally evolve. However, other divergences point to substantive research gaps rather than disciplinary specialization. The weak co-occurrence between studies on mercury exposure and those addressing biodiversity change, landscape degradation, or socio-ecological systems suggests that key cross-scale linkages remain poorly integrated in current research programs. These are precisely the intersections where ecological mechanisms, contaminant pathways, and human vulnerability jointly determine the outcomes of mining impacts in the Amazon.

The absence of integrative bridges in current research limits understanding of how habitat alteration influences mercury uptake, biodiversity loss shapes exposure pathways, and social and territorial contexts mediate community-level risk. Addressing these gaps requires a minimum indicator set capturing contaminant dynamics, ecological responses, and human exposure, e.g., total and methylmercury in water and sediment, sentinel fish species, macroinvertebrate indices, and human biomarkers (Vieira et al. 2018; de Vasconcellos et al. 2022; Moraes et al. 2023; Canela et al. 2024; Escobar-Camacho et al. 2024), with sampling intervals designed to capture both hydrological variability and episodic contamination. Priority research questions include: (i) How do mining-driven landscape changes alter mercury bioavailability and trophic transfer? (ii) Which ecological indicators best predict human exposure gradients? (iii) How do governance regimes and Indigenous protections mediate contamination–ecology linkages? By integrating these approaches, studies can bridge thematic gaps revealed by our topic dissimilarity analysis, enabling cross-scale, interdisciplinary monitoring of mining impacts in the Amazon.

### Temporal Shifts and Socio-Political Drivers of Research Priorities

The gaps verified are mirrored in the temporal evolution of research topics, revealing how the focus and framing of mining-related studies have shifted over the past decades. The temporal divergence among topics reflects not only shifting research priorities but also structural transformations in how mining-related disturbances are conceptualized in Amazonian systems. The long-term decline in contaminant-focused themes suggests that mercury, once a unifying axis of ecological and public-health concern, no longer operates as the dominant integrative framework linking mining, ecosystems, and society. This does not imply reduced importance, but rather a displacement by more complex socio-environmental narratives as researchers increasingly acknowledge the multiplicity of pathways through which mining reorganizes landscapes. The rise of topics centered on Indigenous rights and territorial governance exemplifies this shift; these themes act as new “organizing units” of inquiry, around which ecological, political, and cultural processes become analytically entangled. Such research is in line with recent evidence showing that Indigenous lands mitigate mining-induced deforestation (Ribas and Galetti 2025) and with studies documenting the erosion of Indigenous territorial protections by weak legal enforcement and mining pressures (Mataveli et al. 2022; Oviedo and Senra 2023). These dynamics expand the conceptual boundary of mining impacts beyond simple biophysical degradation, embedding extractive activities in the longer arc of dispossession, land‑use conflict, and governance asymmetry.

At the same time, the irregular patterns in contamination and disease‑related topics reflect the episodic nature of crisis‑driven science, where scholarly attention often contracts and expands in response to socio‑political triggers rather than steady ecological trends. The gradual increase in socio‑ecological and deforestation‑related themes points to a growing recognition that mining must be understood within broader land‑system transitions. Indeed, mining-driven deforestation is deeply embedded in infrastructure expansion, settlement dynamics, and changing tenure regimes (Sonter et al. 2017; Justino et al. 2025). These trajectories imply that the field is moving from a pollutant-centric paradigm toward a systems-level understanding in which mining interacts with hydrological, institutional, and socio-cultural processes in nonlinear ways.

Importantly, the lack of synchronized trends across topics should not be misread as fragmentation. Instead, it reflects the coexistence of research lineages operating at different temporal and conceptual scales, some stabilizing as “mature” domains, others expanding as socio-environmental conditions evolve. This heterogeneity is itself informative; it reveals where scientific attention is path-dependent, where it is crisis-responsive, and where it is structurally reoriented by political and territorial transformations. Such nuances underscore that those temporal dynamics in topic prevalence do not merely track research volume but map the evolving conceptual architecture of how mining is understood as an ecological and societal force in the Amazon.

Furthermore, these evolving research priorities must be interpreted within a dynamic and often adverse regulatory context. Recent political and corporate strategies in Brazil have actively sought to dismantle environmental protections, particularly in sensitive areas like the Border Strip and Indigenous Lands. The mining sector employs a range of tactics, from political-institutional lobbying for bills that reduce the Border Strip (e.g., Brazilian Federal Bill of Law No. 2153/2023 and 3937/2022) to the use of subsidiaries and joint ventures to bypass restrictions on foreign capital. The enactment of Decree No. 11,076/2022 (Brasil 2022), which restructured the governance architecture of the mineral sector and streamlined approval mechanisms, has introduced additional challenges for public oversight. This regulatory flexibilization, coupled with the persistent expansion of illegal mining networks, underscores that the conceptual shifts identified in the literature are a direct response to the accelerating material and political pressures on Amazonian territories. Therefore, future research and monitoring frameworks must be politically literate, designed to track impacts generated by both formal corporate strategies and widespread illicit operations.

### Limitations

Our approach provides a comprehensive synthesis of mining-related ecological research in the Amazon, but several limitations should be acknowledged. First, the LDA model identifies patterns of word co-occurrence, which is valuable for detecting thematic structures but does not capture causal relationships or the full conceptual depth of the topics. The bag-of-words framework ignores word order, is sensitive to synonymy, and may be affected by temporal drift in terminology, which can limit the model’s semantic resolution.

Second, our analysis is restricted to peer-reviewed literature indexed in major databases (Web of Science, Scopus, SciELO), excluding grey literature, governmental reports, and community-based observations that may contain locally relevant knowledge. High dissimilarity values reflect limited co-occurrence in the literature but do not necessarily imply the absence of substantive connections in the real world.

In interpreting these patterns, it is also important to consider the robustness of the topic structure. Although topic stability across multiple random initializations showed only moderate similarity, replicated models consistently converged on the same broad thematic domains. This suggests that heterogeneity in the corpus, spanning decades, disciplines, and methodological approaches, naturally produces some variability, but without compromising the substantive coherence of the 12-topic solution. The combination of high coherence and cross-seed convergence, therefore, provides confidence that the identified thematic patterns reliably reflect how mining impacts in the Amazon have been studied, rather than artifacts of stochastic model variation.

Future studies could address these limitations using more advanced approaches, such as Structural Topic Models (STM), Dynamic Topic Models, or embedding-based methods (e.g., BERTopic), which allow incorporation of temporal dynamics, document-level covariates, and richer semantic representations. Triangulating these approaches with qualitative analyses and expert knowledge could provide a more nuanced and actionable understanding of mining’s ecological and social impacts in the Amazon.

## Final Remarks

This review provides a data-driven synthesis of three decades of research on the ecological impacts of mining in the Amazon, identifying 12 dominant themes across five broad categories: Pollution & Toxicology, Land use, Social–ecological systems, Biodiversity & Ecosystem health, and Monitoring & Assessment. Mercury-related issues and post-mining restoration remain central concerns, reflecting persistent risks to ecosystems and human health.

Despite this focus, critical gaps persist. Integration of ecological, social, and health dimensions is limited, and themes such as environmental monitoring, landscape-scale changes, and socio-ecological interactions remain underrepresented. Temporal trends reveal a gradual shift toward topics like Indigenous rights and deforestation dynamics, highlighting increasing recognition of socio-political and territorial aspects of mining impacts.

Future research should adopt integrative, longitudinal approaches that combine ecological monitoring, socio-health indicators, and remote sensing. Strengthening links between contamination studies, biodiversity assessments, and social vulnerability frameworks will be essential to support evidence-based policy and conservation strategies. By mapping the current state of knowledge and identifying persistent gaps, this synthesis provides a roadmap for advancing a more holistic understanding of mining’s ecological and social consequences in one of the world’s most critical and vulnerable biomes.

## Supplementary information

Supplementary information

Supplementary information

## Data Availability

The complete dataset is provided as Supporting Information. Data and R scripts are also publicly available in Zenodo (10.5281/zenodo.17737403). All files are distributed under an MIT License. The dataset contains only publicly available bibliographic metadata (titles, abstracts, authors, DOIs); therefore, no anonymization procedures were required.
