# Supplementary material for: Ecological Impacts of Mining in the Amazon: Thematic Trends and Research Gaps
Source: Environ Manage. 2026 Feb 20;76(4):112. doi: 10.1007/s00267-026-02403-6 (PMC12923496; doi:10.1007/s00267-026-02403-6)
Supplement: Supplementary file 2 — Supplementary information [file 267_2026_2403_MOESM2_ESM.docx]

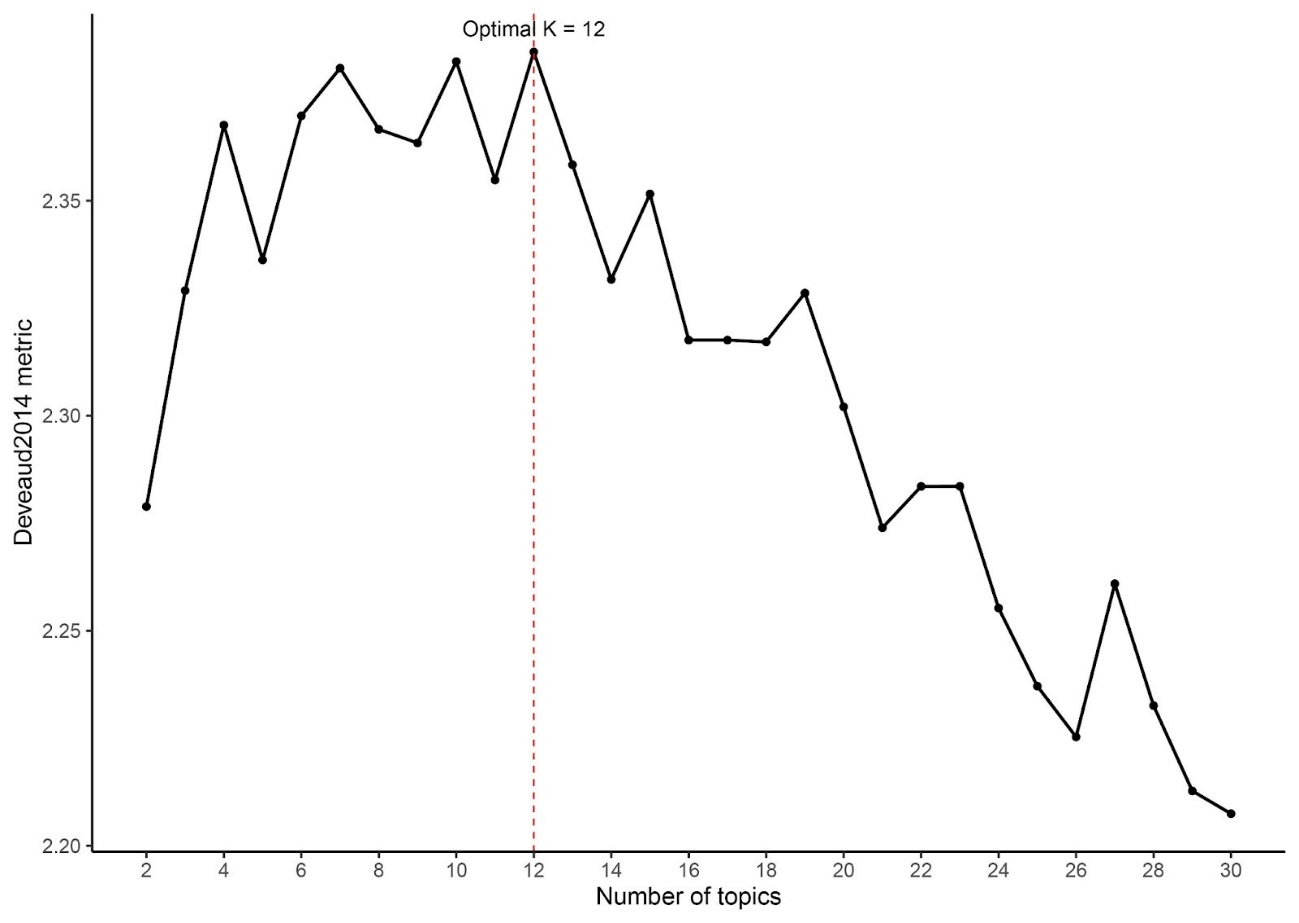
**Fig. S1** Deveaud2014 coherence scores for topic models with k ranging from 2 to 30 (step = 2). The dashed vertical line indicates the optimal number of topics (k = 12), corresponding to the maximum coherence value.


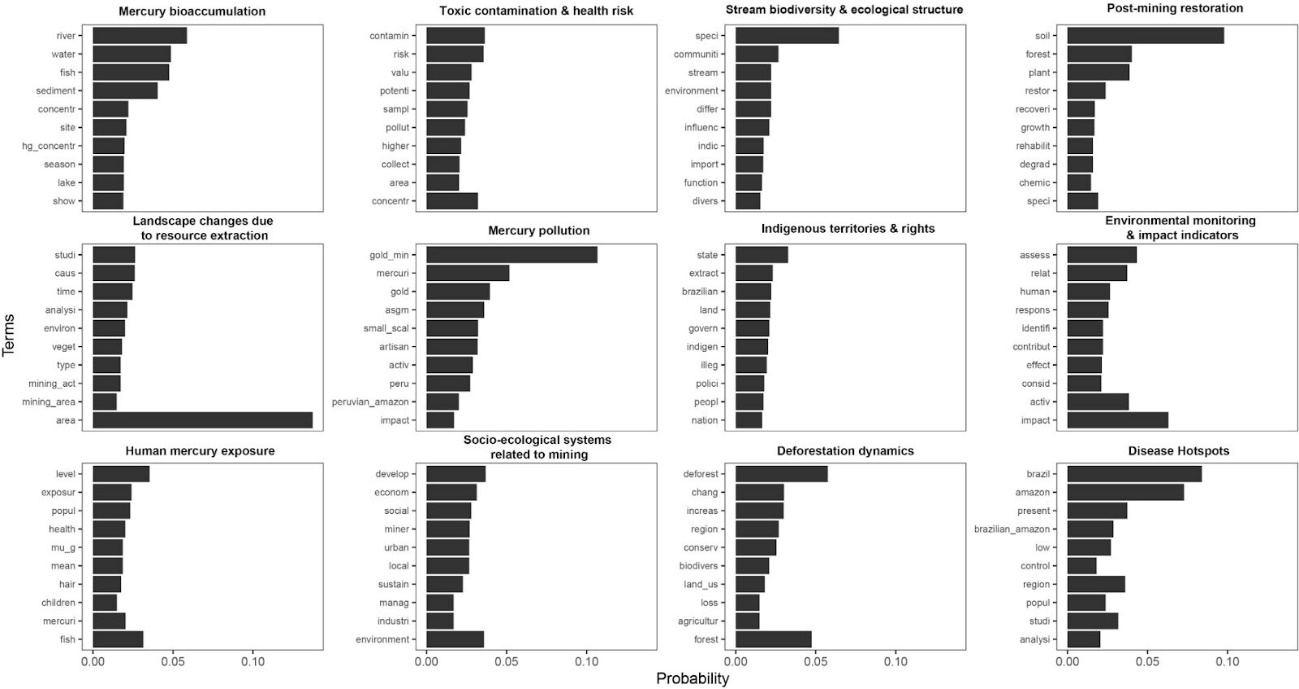


**Fig. S2** Bar plot showing the distribution of the 10 top words in each topic, based on their estimated probabilities (β) in the LDA model
